# Substance use and dietary practices among students attending alternative high schools: results from a pilot study

**DOI:** 10.1186/1471-2458-11-263

**Published:** 2011-04-25

**Authors:** Chrisa Arcan, Martha Y Kubik, Jayne A Fulkerson, Peter J Hannan, Mary Story

**Affiliations:** 1 University of Minnesota, Division of Epidemiology and Community Health, School of, Public Health, 1300 South 2nd Street, Suite 300, Minneapolis, MN 55454, USA; 2 University of Minnesota, School of Nursing, Room 5-140 WDH 1331, 308 Harvard St S E, Minneapolis, MN 55455, USA

## Abstract

**Background:**

Substance use and poor dietary practices are prevalent among adolescents. The purpose of this study was to examine frequency of substance use and associations between cigarette, alcohol and marijuana use and selected dietary practices, such as sugar-sweetened beverages, high-fat foods, fruits and vegetables, and frequency of fast food restaurant use among alternative high school students. Associations between multi-substance use and the same dietary practices were also examined.

**Methods:**

A convenience sample of adolescents (n = 145; 61% minority, 52% male) attending six alternative high schools in the St Paul/Minneapolis metropolitan area completed baseline surveys. Students were participants in the Team COOL (Controlling Overweight and Obesity for Life) pilot study, a group randomized obesity prevention pilot trial. Mixed model multivariate analyses procedures were used to assess associations of interest.

**Results:**

Daily cigarette smoking was reported by 36% of students. Cigarette smoking was positively associated with consumption of regular soda (p = 0.019), high-fat foods (p = 0.037), and fast food restaurant use (p = 0.002). Alcohol (p = 0.005) and marijuana use (p = 0.035) were positively associated with high-fat food intake. With increasing numbers of substances, a positive trend was observed in high-fat food intake (p = 0.0003). There were no significant associations between substance use and fruit and vegetable intake.

**Conclusions:**

Alternative high school students who use individual substances as well as multiple substances may be at high risk of unhealthful dietary practices. Comprehensive health interventions in alternative high schools have the potential of reducing health-compromising behaviors that are prevalent among this group of students. This study adds to the limited research examining substance use and diet among at-risk youth.

**Trial registration number:**

ClinicalTrials.gov: NCT01315743

## Background

Substance use and poor dietary practices are prevalent among adolescents [1,2]. In the general population, substance abuse is strongly correlated with violent behavior, weapon carrying in school, motor vehicle accidents, unintended pregnancy, and HIV [3-9], which are the leading causes of morbidity and mortality in youth. Recent national data indicate that almost one half of adolescents have smoked cigarettes [1] in their lifetime. Among 12^th ^grade students, about 42% have tried marijuana and almost three out of four consumed alcohol in their lifetime [2].

An unhealthful diet can compound the health effects of substance use, since poor diet and substance use are independent risk factors for chronic diseases, such as atherosclerosis and some cancers [10-12]. The majority of adolescents do not have diets that follow the Dietary Guidelines for Americans; close to 80% of adolescents do not consume the daily recommended servings of fruits and vegetables and one third consume regular soda at least one time a day [1]. Observational studies have often found covariation of health risk behaviors among both adolescents [13-17] and adults [15,18-20]. Research on correlations of individual substances and eating behaviors indicates that smoking is associated with greater consumption of fat [21], soft drinks [16], and fast food [22] and with lower consumption of fruits and vegetables [22-25]; alcohol is associated with fat intake [21].

Most studies assessing correlations between substance use and dietary behaviors have done so in traditional high schools [13,21,22,26]. Factor analysis guiding the identification of clusters of behaviors among a large sample of adolescents indicates that adolescents engaging in risk-seeking behaviors, such as tobacco, alcohol and marijuana use have almost twice the odds ratio of unhealthy eating [13]. In a large sample of diverse middle and high school students, smoking frequency was directly related to frequency of fast food consumption and inversely related to regular breakfast, lunch, and dinner consumption [22]. A study by Burk and colleagues examining substance use, physical activity and diet in a sample of 18 year old male and female students found clustering of unhealthy behaviors; both males and females who smoked engaged in unsafe drinking and females had low levels of fiber whereas males had high fat intake [21].

Research shows that at-risk youth who use substances are more likely to be involved in violent behavior, drop-out of school, truancy, and engage in multiple health behaviors [27-30]. One group of adolescents who are at-risk of dropping out of school [31] and are likely to be involved in high-risk behaviors are students who attend alternative high schools. Alternative high schools are more common in urban districts, and roughly two-thirds (62%) of schools enroll more than 50% of minorities and close to one-half enroll greater than 20% of students below the poverty line [31]. A study examining correlations between cigarette smoking and various socioeconomic status (SES) indicators in middle and high school students found that students who attend schools with high percentages of participation in the reduced/low cost lunch program have almost 6 times the odds of smoking than students in schools with low participation in the reduced/low cost lunch program [32]. Previous studies have shown that when compared to traditional high school students, a higher percentage of alternative high students ever tried a substance; currently use substances; and drive under the influence of alcohol [14,33]. A study using national data of alternative high school students found close to one in five students having used multiple substances on school property [33]. A study in a Texas district found 77% of males in an alternative school compared to 20% in a traditional school to have smoked at least one cigarette in the past month. The same study found 85% and 88% of alternative school males and females, respectively reported having five drinks at one time on at least one occasion in the past month, compared to 55% and 34% of males and females in traditional schools [34]. Recent studies have also indicated a high prevalence of overweight and obesity, unhealthful diet, and low physical activity levels among alternative high school students [35-37]. However, to our knowledge, no studies to date have examined correlations between substance use and dietary behaviors among alternative high school students.

Substance abuse, tobacco use and nutrition and overweight are three of the twenty eight focus areas that are addressed by Healthy People 2010 agenda to reach the goal of increasing quality and years of healthy life [9]. Thus, in order to develop comprehensive health education programs and to achieve improved health and longevity, examining correlates of substance use and dietary practices among adolescents is essential. The present study was guided by the ecological model [38] and its goals were to: 1) examine frequency of substance use among a high risk group of youth; 2) assess correlations between cigarette, alcohol, and marijuana use and dietary practices, such as consumption of regular soda, sports drinks, other-sugar sweetened beverages, high-fat foods, fruits and vegetables, and fast food restaurant use; and 3) assess correlations between multi-substance use and the same dietary practices. All analyses were conducted with students attending alternative high schools, a high-risk group of youth.

## Methods

### Study Design and Sample

The present study used a cross-sectional design to examine baseline data from the Team COOL (Controlling Overweight and Obesity for Life) pilot study, a multi-component diet and physical activity intervention trial to promote healthy weight loss or prevent excess weight gain among alternative high school students. Six alternative public high schools (four urban and two suburban) in the St. Paul-Minneapolis, Minnesota were contacted and agreed to participate. The data were collected in fall 2006 before schools were randomized to intervention and control conditions. Additional details about the study design are described elsewhere [39].

Student enrollment across schools varied from 27 to 142 students (mean: 102 students), with an average age of 17.3 years (range: 14.1-19.8 years). All students enrolled in the schools were eligible and were invited to participate in study measurements. Students who were younger than 18 years were given parental consent forms. On the day of measurement, trained study staff collected assents from all students and the signed parental consents from those younger than 18 years old. The students completed a 76-item survey that took about 30 to 40 minutes. The students who completed the survey had their height/weight measured and received a $5 gift card. Across the six schools, a total of 145 students participated in the baseline data collection. There was a high percentage of minority students (including all races except white; mean = 64%: range = 31% to 96%) and students receiving free/low-cost lunch (mean = 61%: range = 40% to 96%). All study procedures were approved by the University of Minnesota's Institutional Review Board Human Subjects Committee. Due to the variable nature of student attendance in alternative high schools, the study participation rate was derived by multiplying the prior year's (2005-2006) attendance rate with the schools' 2006-2007 student enrollment [40], to give an estimated average adjusted enrollment of 68 students (range: 16 to 107). The participation rate across schools was 36% (range: 18% to 100%).

### Measures

The following dependent and independent variables were examined in this study.

### Dependent Variables

#### Regular soda, sports drinks and other sugar-sweetened beverage consumption

Regular soda, sports drinks and other sugar-sweetened beverages (kool-aid, fruit drinks, lemonade or energy drinks) were assessed by asking participants to report frequency of consumption of each type of beverage over the past month [41]. Ten response categories ranged from 'Never' to '5 or more times a day.' For each beverage, data were recoded to times per week and modeled as a continuous variable. Higher values indicated more times per week a beverage was consumed.

#### Fast food restaurant use

Frequency of fast food restaurant use was assessed with the question [42], "Outside of the school day, during a normal week (including weekend days), how many times do you eat or drink something from a fast food restaurant, like McDonald's, Taco Bell or Pizza Hut?" Six response categories ranged from 'Never' to 'More than 7 times.' The data were recoded to represent times per week and modeled as a continuous variable. Higher values indicated more times a week of eating or drinking something from a fast food restaurant.

#### High-fat food intake

The 17-item fat screener developed by Block and colleagues and validated in an adult population was used to assess high-fat food intake [43]. Due to the older average age of the adolescents in this study, the screener was deemed appropriate for use. Examples of high-fat food items included various meats, hot dogs, fried chicken, pizza, whole milk and cheese, French fries, and doughnuts. Five response categories ranged from '1 time a month or less' to '5 or more times a week.' The data were recoded to represent times a week and modeled as a continuous variable. The Cronbach's alpha for the study sample was 0.89. Students whose responses were greater than 3 standard deviations (SD) from the mean were excluded from the analysis (n = 2). Higher values indicated more times a week of high-fat food consumption.

#### Fruit and vegetable intake

Intake of fruits and vegetables was assessed with the 6-item fruit and vegetable screener [44]. The screener was previously validated in a racially and socioeconomically diverse sample of urban high school students [44]. The items included 100% fruit juice, fruits, vegetables, green salad, potatoes (excluding French fries), and carrots. Six response categories ranged from 'Less than once a week' to '5 or more times a day.' Data were recoded as daily servings and modeled as a continuous variable. The Cronbach's alpha for the study sample was 0.85. Students whose responses were greater than 3 SDs from the mean were excluded from the analysis (n = 2). Higher values indicated more servings per day of fruit and vegetable consumption.

### Independent Variables

#### Substance Use

A single item adopted from the Youth Risk Behavior Surveillance System (YRBSS) was used to measure frequency of substance use [45]. Students were asked to report how often they used the following substances during the past year: a) cigarettes, b) beer, wine, hard liquor, c) marijuana, and d) drugs other than marijuana (acid, cocaine, etc). In the current study, only the first three categories were used since the majority of students reported never having used drugs other than marijuana. The measure demonstrated good reliability [45] and has been used in other studies with adolescents [22]. The response categories were 'never', 'a few times', 'monthly', 'weekly', or 'daily.' To describe frequency of substance use, the categories were collapsed to 'never', 'frequent, but not daily use', and 'daily use.'

To examine associations between frequency of each substance and dietary practices, each substance was used as a continuous variable. We also examined associations between multi-substance use and dietary behaviors. The multi-substance use variable was created by first dichotomizing the original responses of each substance to 'never' or 'ever' having used each substance during the past year. A four-category multi-substance use variable was then created by summing the newly created dichotomized substance use variables (cigarette, alcohol, and marijuana). The four categories representing multi-substance use in the past year included: 1) never used any substance, 2) used any one substance, 3) used any two substances, and 4) used all three substances. We also assessed the overall impact of substance use on diet quality by treating the six dietary behaviors as correlated outcomes within individual. The repeated measures analysis of the six dietary behaviors estimated a single association of substance use on the overall diet quality. All sugar-sweetened beverages, high-fat food, and fast food restaurant use were reverse coded to represent a healthful diet quality.

#### Demographic Characteristics

Gender, age, race/ethnicity and SES were included in the models as potential confounders. Age and gender were obtained from school records; age was modeled as a continuous variable. Students reported their race/ethnicity and categories included: a) American Indian or Alaskan Native, b) Asian (including Cambodian, Hmong, Korean, Laotian, and Vietnamese), c) Black or African American, d) Hispanic or Latino, e) White, and f) Other." To ensure adequate sample size for analyses, the categories were collapsed to White, Black and other. The 'other' racial/ethnic category included the following groups: American Indian or Alaskan Native (1%); Asian, including Cambodian, Hmong, Korean, Laotian, and Vietnamese (6%); Hispanic or Latino (9%); multi-ethnic non-Hispanic (10%); other (3%). For most students, SES was measured with the question "Do you get free/low-cost lunches at school?" (n = 135). Response categories were 'Yes', 'No', and 'I don't know.' If the response was missing or 'I don't know,' the question 'Does your family get public assistance (welfare, food stamps or other assistance?) was used (n = 8) [46]. A 'Yes' response indicated lower SES and a 'No' response indicated higher SES.

### Statistical Analysis

Descriptive statistics were used to assess the frequency of cigarette smoking, alcohol and marijuana use and the frequency of multi-substance use by demographic characteristics in our sample of alternative high school students. Chi-square and t-tests were used to test bivariate associations between substance use and demographic characteristics. Mixed model analysis of variance was used to assess the association between each substance and each dietary practice. Mixed model analysis of variance was also used to examine associations between multi-substance use (four-category variable) and dietary practices. Separate analyses were conducted for each dietary practice, and each analysis was controlled for gender, race/ethnicity, age, and SES.

Since student intake of each type of sugar-sweetened beverage and fruits and vegetables were positively skewed, they were square-root transformed in all mixed model analyses when determining statistical significance. The square-root transformed variables are approximately Gaussian distributed, thus the p-values using the transformed variables are more accurate since they are dependent on the tails of the distribution. However for ease of interpretation, the presentation of mean servings was generated on the natural scale (untransformed). The school variable was included in the model as a random effect, accounting for the additional component of variance associated with a cluster sampling design where observations from students from the same schools may be correlated [47]. PROC MIXED procedures were utilized since the outcome variables were continuous. The level of significance was set at two-sided p < 0.05.

## Results

Table 1 shows frequencies of each substance used by socio-demographic characteristics. Overall, 36% of students reported daily use of cigarettes, 13% marijuana and 3% alcohol. Females and males reported equal percentages of daily cigarette (36%) and marijuana (13%) use. White students and those in higher SES had higher daily consumption of each substance compared to their counterparts. Table 2 depicts frequency of multi-substance use by socio-demographic characteristics. Overall, 79% of the students used at least one substance and almost one-third used all three substances in the past year.

**Table 1:** Frequency of substance use among alternative high school students by demographic characteristics in the past 12 months (n = 145)^a^

	Cigarette Smoking			Alcohol Use			Marijuana Use		
**Demographic** **Characteristics**	**Never ** **n (%)**	**Frequent, but** **not daily use** **n (%)**	**Daily use** **n (%)**	**Never** **n (%)**	**Frequent, but** **not daily use** **n (%)**	**Daily use** **n (%)**	**Never** **n (%)**	**Frequent, but** **not daily use** **n (%)**	**Daily use** **n (%)**

Total (N = 145)	60 (42%)	32 (22%)	52 (36%)	60 (42%)	80 (55%)	4 (3%)	74 (51%)	52 (36%)	19 (13%)
Gender	(n = 144)			(n = 144)			(n = 145)		
Male	33 (44%)	15 (20%)	27 (36%)	29 (39%)	43 (57%)	3 (4%)	38 (50%)	28 (37%)	10 (13%)
Female	27 (39%)	17 (25%)	25 (36%)	31 (45%)	37 (54%)	1 (1%)	36 (52%)	24 (35%)	9 (13%)
Race	(n = 144)			(n = 144)			(n = 145)		
Black	26 (58%)	11 (24%)	8 (18%)	27 (60%)	17 (38%)	1 (2%)	24 (52%)	14 (30%)	8 (14%)
White	16 (28%)	11 (19%)	30 (53%)	16 (28%)	39 (68%)	2 (4%)	28 (49%)	21 (37%)	8 (17%)
Other	18 (43%)	10 (24%)	14 (33%)	17 (41%)	24 (57%)	1 (2%)	22 (52%)	17 (40%)	3 (7%)
SES	(n = 142)			(n = 142)			(n = 143)		
Higher	16 (31%)	11 (22%)	24 (47%)	17 (33%)	32 (63%)	2 (4%)	24 (46%)	20 (38%)	8 (15%)
Lower	42 (46%)	21 (23%)	28 (31%)	41 (45%)	48 (53%)	2 (2%)	48 (53%)	32 (35%)	11 (12%)
Age	(n = 144)			(n = 144)			(n = 145)		
Mean (SD)	16.9 (1.3)	17.4 (1.1)	17.4 (0.97)	16.8 (1.2)	17.5 (1.0)	17.7 (0.63)	17.1 (1.2)	17.4 (1.0)	17.1 (1.2)

^a ^Cases in each analysis ranged from 142-145 due to incidental missing data. 'Frequent but not daily use' indicates those who reported using a substance at least few times in the past 12 months but less often than daily; 'daily use' indicate those who reported using a substance daily

**Table 2:** Multi-substance use among alternative high school students by socio-demographic characteristic in the past 12 months

	**Number of Substances Used in the Past 12 Months **^**a**^				
**Demographic Characteristics**	**Never Used Any** **n (%)**	**Used any one** **n (%)**	**Used any two** **n (%)**	**Used all three** **n (%)**	**P-Value**

Total (n = 145)	31 (21%)	33 (23%)	37 (26%)	44 (30%)	
Gender					
Male (n = 76)	17 (22%)	18 (24%)	15 (20%)	26 (34%)	0.389 ^b^
Female (n = 69)	14 (20%)	15 (22%)	22 (32%)	18 (26%)	
Race/Ethnicity					
White (n = 57)	8 (14%)	10 (18%)	16 (28%)	23 (40%)	0.095 ^b^
Black (n = 46)	14 (31%)	12 (26%)	13 (28%)	7 (15%)	
Other (n = 42)	9 (22%)	11 (26%)	8 (19%)	14 (33%)	
SES					
Higher (n = 52)	9 (17%)	8 (15%)	16 (31%)	19 (37%)	0.251 ^b^
Lower (n = 91)	20 (22%)	25 (27%)	21 (23%)	25 (28%)	
Age (n = 145)					
Mean (SD)	16.7 (1.3)	17.1 (1.2)	17.3 (1.1)	17.6 (1.0)	0.032 ^c^

^a ^Substances include cigarette smoking, alcohol, and marijuana use in the last 12 months. 'Never used any' indicate those who never used any of the three substances in the last 12 months; 'Used only one', those that used any one substance; 'Used any two', those that used any two substances; 'Used all three', those that used all three substances in the last 12 months.

^b ^χ^2 ^test of independence of substance use and demographic characteristics.

^c ^t-test of independence of substance use and student age.

Table 3 presents the results of the multivariate models assessing associations between each substance and each dietary practice, adjusted for potential confounders. Cigarette smoking was positively associated with consumption of regular soda (p = 0.019), high-fat foods (p = 0.037), and fast food restaurant use (p = 0.002). For example, higher use of cigarette smoking was associated with an additional weekly consumption of 1.22 times of regular soda. Alcohol use (p = 0.005) and marijuana use (p = 0.035) were positively associated with high-fat food consumption. All other estimated associations were statistically non-significant at the p<0.05.

**Table 3:** Multivariate associations between each substance and selected dietary practices in alternative high school students in the past 12 months (N = 145)

	Substance use in the past 12 months ^a^								
**Dietary Intake**	**Cigarette Smoking** **(n = 144)**			**Alcohol Use** **(n = 144)**			**Marijuana Use** **(n = 145)**		

	**Estimate (b)**	**95% CI**	**p-value**	**Estimate (b)**	**95% CI**	**p-value**	**Estimate (b)**	**95% CI**	**p-value**
Regular soda times/wk	1.22	(0.25,2.2)	0.019	0.79	(-0.78,2.39)	0.295	1.05	(-0.14,2.25)	0.093
Sports drinks times/wk	-0.08	(-0.79,0.63)	0.972	0.03	(-1.12,1.20)	0.847	-0.41	(-1.28,0.45)	0.838
Other sugar-sweetened beverages times/wk	0.77	(- 0.05,1.61)	0.071	1.34	(0.02,2.67)	0.079	-0.08	(-1.10,0.94)	0.893
Fast food restaurant times/wk	0.26	(0.09,0.42)	0.002	0.18	(-0.09,0.46)	0.194	0.10	(-0.10,0.30)	0.329
High-fat foods times/wk	1.24	(0.07,2.41)	0.037	2.61	(0.76,4.45)	0.005	1.51	(0.10,2.92)	0.035
Fruits/vegetables servings/day	0.11	(-0.30,0.53)	0.498	-0.33	(-1.00,0.33)	0.463	-0.32	(-0.82,0.18)	0.287

^a ^All estimates are adjusted for age, gender, race, and SES

Table 4 presents adjusted means of dietary practices by categories of multi-substance use. Multi-substance use was significantly associated with high-fat food intake (p = 0.0003). Students who used all three substances over the past year consumed high-fat foods 11 additional times per week than students who never used any substance. Also, those who used any one substance or any two substances had additional weekly consumption of 12 and 7 times of high-fat foods, respectively, compared to those who never used any substance (p-value for trend = 0.002). Regular soda consumption and multi-substance use were marginally positively associated (p = 0.058). Specifically, there was an additional weekly consumption of 5.4 times of regular soda between those who used all three substances and those who did not use any substance in the past year (p-value for trend = 0.008). There were no significant associations between categories of multi-substance use and fruit and vegetable intake.

**Table 4:** Mean dietary practices among alternative high school students by category of multi-substance use (N = 145)

	Number of Substances Used in the Past 12 Months *				
Dietary Practices	Never Used Mean (SE) n = 31	Used any one Mean (SE) n = 33	Used any two Mean (SE) n = 37	Used all three Mean (SE) n = 44	P Value†
Regular soda times/wk	7.15 (1.8)	7.42 (1.7)	12.08 (1.6)	12.59 (1.5)	0.058
Sports drinks times/wk	2.99 (1.7)	4.26 (1.6)	6.00 (1.5)	2.27 (1.4)	0.307
Other sugar-sweetened beverages times/wk	5.01 (1.8)	7.28 (1.8)	8.44 (1.7)	8.34 (1.6)	0.400
Fast food restaurant times/wk	2.05 (0.33)	3.04 (0.3)	2.93 (0.2)	2.92 (0.2)	0.113
High-fat foods times/wk	18.23 (2.2) ^a^	30.07 (2.1) ^b^	25.41 (2.0) ^b^	29.17 (1.9) ^b^	0.0003
Fruits/vegetables servings/day	2.94 (0.8)	3.82 (0.8)	4.21 (0.7)	2.98 (0.7)	0.728

Sample size may vary across models due to missing responses. Mean values were adjusted for gender, race/ethnicity, SES, and age. R^2 ^for base model ranged from 0.01 for fruits and vegetables to 0.19 for other sugar-sweetened beverages.

* Substances include cigarette smoking, alcohol, and marijuana use in the last 12 months. 'Never used any' indicate those who never used any of the three substances in the last 12 months; 'Used only one', those that used any one substance; 'Used any two', those that use any two substances; 'Used all three', those that used all three substances in the last 12 months.

† P value represents testing for differences in adjusted means of dietary practices by categories of multiple substance use (df = 3).

⁪ Include kool-aid, fruit drinks, lemonade or energy drinks.

^ab ^Different superscripts indicate statistically significant differences between mean dietary practices by category of substance use.

The correlation matrix between the components (dietary practices) of the diet revealed highest correlations between fast food restaurant use and high-fat food intake (0.658) and between sports drinks and other sugar-sweetened beverages (0.595). Consumption of fruits and vegetables were negatively correlated with sports drinks, other sugar-sweetened beverages, high-fat food, and fast food restaurant use (Table 5). There was a significant association (p = 0.010) between multi-substance use and overall diet quality averaged over the six components of the diet. The least squares means and differences indicated that the category of 'no substance use' was positively associated with a diet quality (beta = 0.224) indicating that never having used any substance was related to a healthful diet, including higher intake of fruits and vegetables, and lower intake of sugar-sweetened beverages, high fat foods, and fast food restaurant use. In contrast, any other category of multi-substance use was related to an unhealthful diet (beta range: -0.10 to -0.02) (Data not shown).

**Table 5:** Correlation matrix between the components (dietary practices) of the overall diet quality ^a ^of alternative high school students

Components of the diet quality (dietary practices)	Regular soda times/wk	Sports drinks times/wk	Other sugar-sweetened beverages times/wk	Fast food restaurant times/wk	High-fat foods times/wk	Fruits/vegetables servings/day
Regular soda times/wk	1.0					
Sports drinks times/wk	0.237	1.0				
Other sugar-sweetened beverages times/wk	0.331	0.595	1.0			
Fast food restaurant times/wk	0.64	0.373	0.193	1.0		
High-fat foods times/wk	0.424	0.251	0.395	0.658	1.0	
Fruits/vegetables servings/day	0.139	-0.515	-0.387	-0.305	-0.667	1.0

^a ^The overall impact of substance use on diet quality was assessed by treating the six dietary behaviors as correlated outcomes within individual. The repeated measures analysis of the six dietary behaviors estimates a single association of substance use on the overall diet quality. All sugar-sweetened beverages, high-fat food, and fast food restaurant use were reverse coded to represent a healthful diet quality.

## Discussion and Conclusions

This study described frequency of cigarette, alcohol, marijuana and multi-substance use and associations between each substance as well as multi-substance use and dietary practices, such as such as sugar-sweetened beverages, high-fat foods, fruits and vegetables, and frequency of fast food restaurant use among alternative high school students. Our results revealed that cigarette was the substance most frequently used by both males and females. Cigarette, alcohol and marijuana use were each associated with higher consumption of high-fat foods, and cigarette smoking was associated with higher consumption of regular soda and fast food restaurant use. Students who used multiple substances had progressively higher consumption of high-fat foods compared to nonusers and single substance users.

Daily smoking was more prevalent among this sample of alternative high school students (36%) compared to a large socioeconomically and racially diverse sample of traditional high school students (14%) [22] and to national surveillance data (10%) [2].

Daily marijuana use among students was twice as high (13%) as among traditional high school students participating in national surveys (6%) [2]. In contrast, annual frequency of alcohol consumption in this sample (59%) was similar to national surveillance data (61%) [2]. The high prevalence of substance use in this study sample was also observed in other studies of alternative high school students [48-50]. According to the 2007 Minnesota Student Survey Alternative High Schools, frequency of any use of cigarette, alcohol or marijuana in the past 30 days was 61%, 57%, and 47%, respectively [50]. In contrast, statewide data of students attending traditional high schools indicated a rate for cigarette, alcohol, and marijuana use over the same period of 25%, 47%, and 20%, respectively [51].

Although studies show that frequency of substance use is higher among low compared to high SES populations, in our study it appears that the opposite was true. However, in our study, SES was labeled as 'lower' or 'higher' to indicate relative SES because of high prevalence of low income students in alternative high schools. Therefore, the labels do not denote 'high' or 'low' as this is true in other studies that measure SES with reported family income. The fact that in our study 'higher SES' students used slightly more substances than 'lower SES' may indicate that' higher SES' students might have the means to purchase these substances. Overall, the findings of this study agree with the literature indicating that alternative high school students smoke cigarettes and use marijuana more frequently compared to high school students participating in national studies.

Direct comparison of the frequency of multi-substance use cannot be made with other data, because of measurement differences [26]. However our results support the covariation of health-compromising behaviors as indicated by the high frequency of multi-substance use and its association with high-fat food intake among the students in this study. A study examining nutrition-related behaviors and categories of substance use (high-risk, conventional, and abstainers) among 11-12 graders from an urban school district found high-risk substance users (combined use of PCP, heroin, crack/cocaine, cigarette, alcohol, or marijuana) to be eating at fast food and convenience stores more frequently than all the other groups [26]. Also, the association between cigarette smoking and consumption of regular soda, high-fat food, and fast food restaurant use in this sample is in agreement with other studies involving adolescents [16,21,22,52]. A study of a large racially and socioeconomically diverse group of adolescents showed that those who smoked had higher soda and fast food consumption [22].

In a separate analyses for each substance, we also found associations between alcohol and marijuana use and high-fat food intake. A study using data from a statewide survey also found use of cigarette, alcohol, and marijuana to be individually associated with an unhealthful diet among middle and high school students [13]. Previous studies with adolescents have reported inverse associations of cigarette smoking with fruit and vegetable consumption [22-25], but our findings did not support these associations, which may partly be due to measurement differences. In the present study, the 6-item questionnaire was used to measure usual intake of fruits and vegetables over the past year. Compared with the 24-hour dietary recalls, the 6-item questionnaire performed equally to the Harvard Food Frequency Questionnaire [43]; however the lack of variability in fruit and vegetable intake by the students in the current study and the relative small sample may not have been sufficient to detect significant differences in dietary practices by each category of substance use.

One of the strengths of this study was its diverse sample of adolescents with respect to gender, race/ethnicity and SES. The measures of dietary practices and substance use have been previously tested in other adolescent populations. This study is the first to examine associations between categories of multiple substance use and dietary practices among alternative high school students. Although the student participation rate was lower than desired, the demographic distribution of our sample closely resembles the demographic characteristics of the students in the study schools (male = 51%; black = 42%, white = 39%; low SES = 56%). A limitation of the study may be the introduction of selection bias. Although all students were invited to participate in the measurements, students who were absent or decided not to participate may differ from the ones who participated, in regard to substance use and dietary practices. The study utilized a cross-sectional analysis that only considers the associations between correlate and outcome variables. Given the relatively small sample size and that participants in this study represent only the Twin Cities area of Minnesota, the generalizability of the findings is limited.

In conclusion, consistent with findings from other studies with alternative high school students, our findings support the high rate of co-occurrence of health-compromising behaviors. As previous studies indicate, behavioral risk factors that contribute to chronic disease begin in adolescence and continue into young adulthood [16,53-55], indicating that the age of initiation significantly predicts substance use and frequency later in life. For example, consistent positive associations have been observed between alcohol use in adolescence and smoking in young adulthood and between younger age of smoking initiation and increased smoking frequency in young adulthood [56,57]. Although alternative high school students are particularly vulnerable to engaging in behaviors that compromise their health, studies about substance use and dietary practices among this population are few. Findings from this research will contribute to the limited body of literature regarding the link between substance use and dietary practices and can set the stage for larger studies on co-variation of health compromising behaviors among alternative high school students. Alternative high schools are ideal settings to reach at-risk youth at an early age, who will greatly benefit from comprehensive and targeted health interventions.

## Competing interests

The authors declare that they have no competing interests.

## Authors' contributions

CA collected data, analyzed and interpreted the data, and led the writing and revisions of the manuscript. MYK conceptualized the study, collected data, assisted with data interpretation and critically edited the manuscript. JAF collected data, assisted with data analysis and critically edited the manuscript. PJH assisted with data analysis and critically edited the manuscript. MS assisted in the conceptualization of the study and critically edited the manuscript. All authors read and approved the final manuscript.

## Pre-publication history

The pre-publication history for this paper can be accessed here:

http://www.biomedcentral.com/1471-2458/11/263/prepub
